# A Method for Functional Trans-Complementation of Intracellular *Francisella tularensis*

**DOI:** 10.1371/journal.pone.0088194

**Published:** 2014-02-04

**Authors:** Shaun Steele, Sharon Taft-Benz, Thomas Kawula

**Affiliations:** Department of Microbiology and Immunology, University of North Carolina at Chapel Hill, North Carolina, United States of America; University of Louisville, United States of America

## Abstract

*Francisella tularensis* is a highly infectious bacterial pathogen that invades and replicates within numerous host cell types. After uptake, *F. tularensis* bacteria escape the phagosome, replicate within the cytosol, and suppress cytokine responses. However, the mechanisms employed by *F. tularensis* to thrive within host cells are mostly unknown. Potential *F. tularensis* mutants involved in host-pathogen interactions are typically discovered by negative selection screens for intracellular replication or virulence. Mutants that fulfill these criteria fall into two categories: mutants with intrinsic intracellular growth defects and mutants that fail to modify detrimental host cell processes. It is often difficult and time consuming to discriminate between these two possibilities. We devised a method to functionally trans-complement and thus identify mutants that fail to modify the host response. In this assay, host cells are consistently and reproducibly infected with two different *F. tularensis* strains by physically tethering the bacteria to antibody-coated beads. To examine the efficacy of this protocol, we tested phagosomal escape, cytokine suppression, and intracellular replication for *F. tularensis* Δ*ripA* and Δ*pdpC*. Δ*ripA* has an intracellular growth defect that is likely due to an intrinsic defect and fails to suppress IL-1β secretion. In the co-infection model, Δ*ripA* was unable to replicate in the host cell when wild-type bacteria infected the same cell, but cytokine suppression was rescued. Therefore, Δ*ripA* intracellular growth is due to an intrinsic bacterial defect while cytokine secretion results from a failed host-pathogen interaction. Likewise, Δ*pdpC* is deficient for phagosomal escape, intracellular survival and suppression of IL-1β secretion. Wild-type bacteria that entered through the same phagosome as Δ*pdpC* rescued all of these phenotypes, indicating that Δ*pdpC* failed to properly manipulate the host. In summary, functional trans-complementation using bead-bound bacteria co-infections is a method to rapidly identify mutants that fail to modify a host response. *Francisella tularensis* is a facultative intracellular bacterial pathogen and is the causative agent of the disease tularemia. *F. tularensis* enters host cells through phagocytosis, escapes the phagosome, and replicates in the host cell cytosol while suppressing cytokine secretion [1]–[4]. Although substantial progress has been made in understanding the intracellular life cycle of *F. tularensis*, the *F. tularensis* proteins responsible for manipulating many host cell pathways are unknown. Identifying novel host-pathogen effector proteins is difficult because there is no rapid method to reliably distinguish between bacterial proteins that modify host processes and proteins that are involved in bacterial processes that are required for the bacteria to survive or replicate in the intracellular environment. The ability to identify mutants that are deficient for host-pathogen interactions is important because it can aid in prioritizing the investigation of genes of interest and in downstream experimental design. Moreover, certain mutant phenotypes, such as decreased phagosomal escape, hinder investigation of other potential phenotypes. A method to specifically complement these phenotypes would allow for further characterizations of certain *F. tularensis* mutants. Thus we sought to develop a method to easily identify and functionally complement mutants that are deficient for interactions with the host.

## Introduction

In order to distinguish whether a phenotype results from a host-pathogen interaction or an intrinsic bacterial defect, we devised a method to functionally complement and thus identify host-pathogen interactions *in trans*. Wild-type and a mutant strain were tethered to the same magnetic bead to ensure that both bacteria enter the same eukaryotic cell. Since cells are consistently infected with both strains of bacteria via the same phagosome, the wild-type bacteria functionally complement the host-pathogen interactions that the neighboring mutant strain fails to initiate. For example, a mutant deficient for phagosomal escape that co-infects a host cell with wild-type bacteria will escape the phagosome because the wild-type bacteria secrete the effectors required for phagosomal escape. Bacterial mutants that exhibit a phenotype caused by intrinsic deficiencies such as defective metabolite production or acquisition will not be functionally complemented by this method since intrinsic defects cannot be trans-complemented by neighboring bacteria.

To demonstrate the efficacy of this protocol, we functionally complemented cytokine suppression, phagosomal escape, and intracellular survival in *F. tularensis* subsp. *holartica* live vaccine strain (LVS) Δ*pdpC* and Δ*ripA*. The *pdpC* gene is located in the *Francisella* pathogenicity island (FPI), which is proposed to encode a secretion and effector system that facilitates phagosomal escape [5]–[13]. PdpC contributes to phagosomal escape, intracellular survival and cytokine suppression [5], [13]. We therefore used Δ*pdpC* as a model of a mutant that contributes to a host-pathogen interaction.

Δ*ripA* can escape the phagosome but is defective for intracellular growth and cytokine suppression [2], [14]. Δ*ripA* replication is reduced in defined media at a pH of 7.5 compared to a pH of 6.5, which implies that cytosolic pH, rather than a host-pathogen interaction, is responsible for decreased intracellular proliferation [15]. Furthermore, RipA regulates the activity of LpxA, a protein required for *F. tularensis* lipid A synthesis (our unpublished data). The Δ*ripA* strain does not proliferate within host cells, but Δ*ripA* strains encoding *lpxA* suppressors are able to replicate within host cells (our unpublished data). These data imply that Δ*ripA* fails to replicate inside of host cells due to irregular regulation of lipid A synthesis and therefore intracellular proliferation of this mutant should not be restored by co-infection with wild-type organisms.

## Results

### Two bacterial strains consistently bind to the same bead

**Figure 1:**
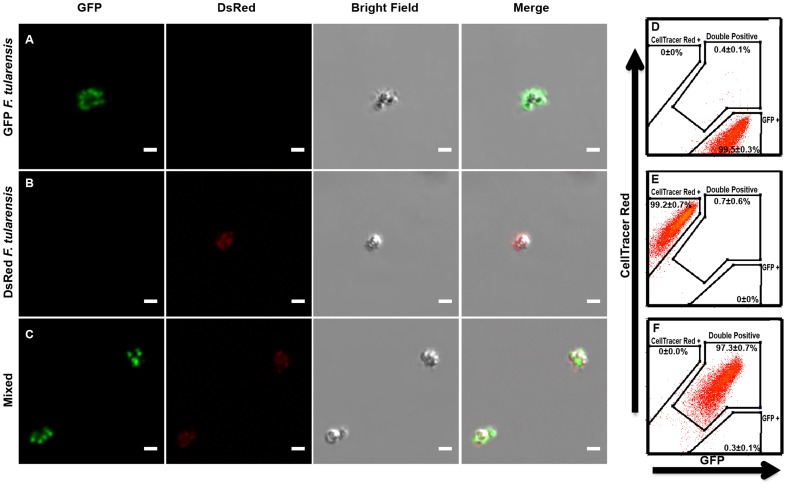
Multiple *F. tularensis* bacteria reliably bind to the same bead. Representative flourecence micrographs of beads bound to **(A)** GFP LVS, **(B)** DsRed LVS, or **(C)** a mixture of GFP LVS and DsRed LVS. All scale bars represent 2 µm. Representative flow cytometry histograms of three independent experiments depicting beads bound to **(D)** GFP LVS, **(E)** CellTrace Far Red labeled LVS, or **(F)** a one to one mixture of GFP LVS and CellTrace Far Red labelled LVS. Histograms were pregated on size to exclude aggregates and non-specific events. Quantification was compiled from all 3 experiments and represents the mean +/− the standard deviation.

Reliable functional complementation of mutants within infected cells requires that both the mutant and complementing strain consistently enter host cells together. To achieve this result we tethered two different *F. tularensis* strains to individual magnetic beads. To test if the two different strains consistently bound to the same bead, we combined anti- *F. tularensis* lipopolysaccharide (LPS) antibody coated beads with a 1∶1 mixture of *F. tularensis* expressing either GFP or DsRed. By microscopy, virtually every observed bead had GFP and DsRed bacteria bound to it (Figure 1 A, B, C). We quantified the amount of beads that bound to both GFP and CellTrace Far Red labeled *F. tularensis* by flow cytometry and found that 97.3+/−0.7% (mean +/− SD) of the beads bound to both GFP and CellTrace Far Red labeled bacteria (Figure 1 D, E, F). This indicates that in infection experiments where wild-type and mutant bacteria are bound to beads, the majority of cells will be infected with both bacterial strains.

Although this method requires multiple bacteria to be present on each bead, too many bacteria infecting the same cell may skew results or phenotypes compared to a normal infection. To quantify the number of bacteria per bead, *F. tularensis* LVS containing a luciferase plasmid was bound to beads and the amount of luminescence per bead was compared to a standard curve. The median bacteria per bead was 3.43 (SEM = 6.69, 4 independent experiments). Additionally, 6.66+/−2.52 intracellular wild-type bacteria per cell were present at 4 hours post inoculation (average +/− SEM, n = 23 from 9 independent experiments, assumptions described in materials and methods). Taken together, we estimate that the average cell is infected with 3 to 8 bacteria using bead co-infections.

### *F. tularensis* is transiently linked to the bead

**Figure 2:**
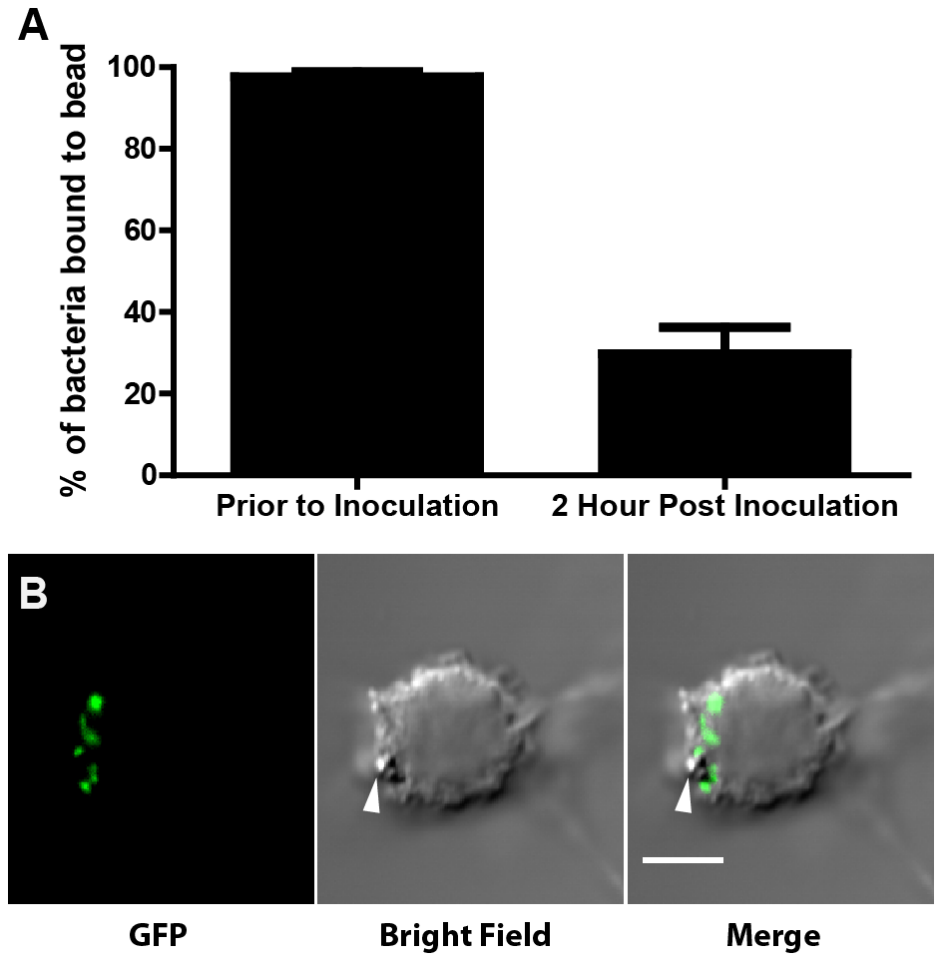
*F. tularensis* separates from the beads inside host cells. **(A)** Percentage of events representing bacteria that were bound to a bead prior to infection (n = 3) and the percentage of events representing intracellular bacteria bound to a bead 2 hours post inoculation (n = 9), as quantified by flow cytometry. Bar graph represents the mean +/− the standard deviation. **(B)** Representative fluorscence micrograph of a cell infected with beads bound to GFP (green) expressing LVS. The white arrow indicates the location of the bead. The image was taken 4 hours post inoculation. The scale bar represents 5 uM.

Tethering the bacteria to the same bead ensures that cells are co-infected with different strains, but irreversible binding of *F. tularensis* to the bead could affect the intracellular life cycle of *F. tularensis*. Thus, we took advantage of a binding mechanism that should allow the bacteria to detach from the bead over time by linking the bacteria to beads coated with antibodies to *F. tularensis* LPS. Each bacterium should initially link to the bead by binding to several anti-LPS antibodies, which should create a high avidity between the bead and the bacterium. The advantage of binding *F. tularensis* to the bead by an anti- LPS antibody is that Gram-negative bacteria, including *F. tularensis*, shed LPS. Thus, viable *F. tularensis* should detach from the bead over time. Indeed, the majority of bacteria were bound to beads immediately prior to infection as determine by flow cytometry (Figure 2A). Intracellular bacteria dissociated from beads within 2 hours during an infection of J774A.1 macrophage-like (J774) cells, presumably due to the bacteria shedding LPS (Figure 2A). Furthermore, microscopy of cells infected with GFP LVS bound to beads shows that some bacteria are spatially separated from the bead by 4 hours post inoculation (Figure 2B). These data demonstrate that the described methodology results in bacteria bound to beads upon initial infection, but that the bacteria detach from the bead following host cell entry. The bacteria also released from beads at similar rates when left in PBS for 2 hours, suggesting that bacterial release from the bead is not mediated by infection (data not shown).

### Bead co-infections functionally complement cytokine suppression

*F. tularensis* suppresses host cell production or secretion of several different cytokines, including interleukin 1β (IL-1β) by both active (such as MAPK inhibition) and passive mechanisms (such as via LPS modifications) [2], [4], [5], [16]–[18]. Since wild-type *F. tularensis* actively suppresses inflammatory responses, co-infection of cells with wild-type bacteria should complement a mutant that fails to suppress the immune response. To test the efficacy of bead co-infections on rescuing immune suppression, we co-infected murine bone marrow derived macrophages (mBMDM) with wild-type *F. tularensis* bound to beads with either Δ*pdpC* or Δ*ripA* and measured IL-1β secretion.

**Figure 3:**
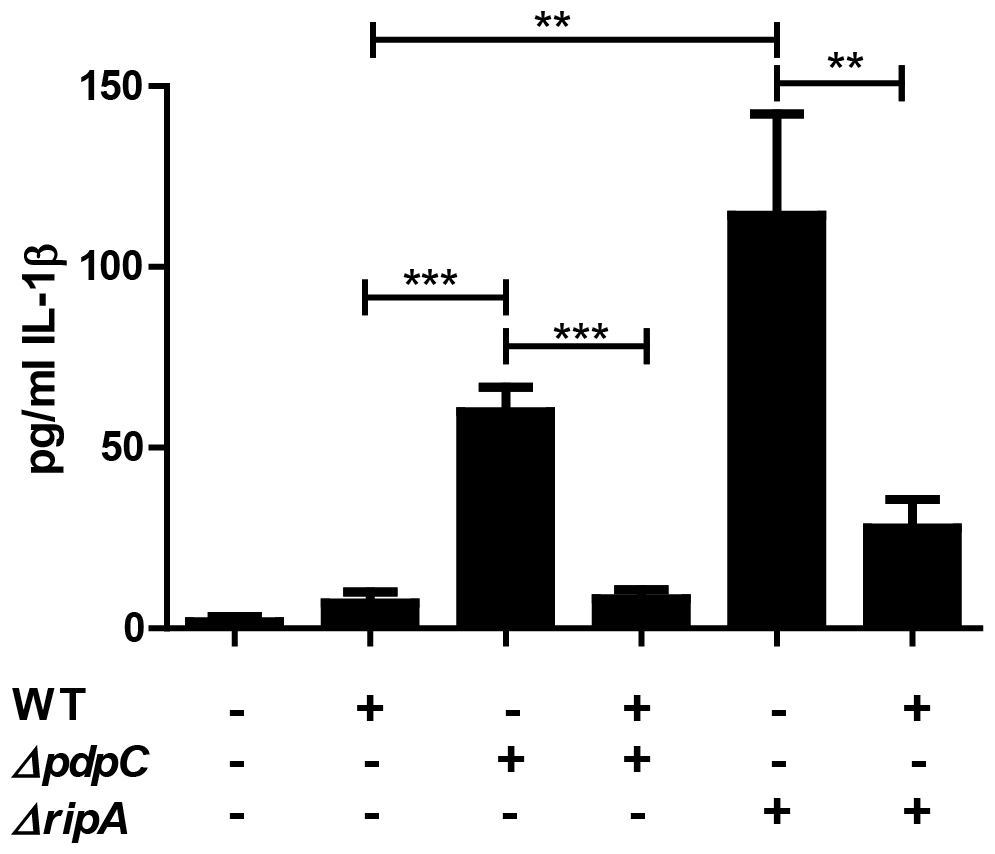
Functional trans-complementation via bead-bound bacteria complements suppression of cytokine secretion. IL-1β ELISA of murine bone marrow derived macrophages that were inoculated with single or mixed inoculations of wild-type, Δ*pdpC* or Δ*ripA* LVS (triplicates, n = 3). All samples were bound to beads prior to infection. Bar graph represents the mean +/− SEM. **p<.01, ***p<.001

Infections of mBMDMs with Δ*ripA* results in increased IL-1β secretion compared to wild-type [2]. Similarly, cells infected with Δ*ripA* bound to beads also induced increased IL-1β secretion compared to cells infected with bead-bound wild-type *F. tularensis* (Figure 3). However, co-infecting cells with wild-type bacteria and Δ*ripA* resulted in reduced IL-1β secretion compared to Δ*ripA* alone (Figure 3). Δ*ripA* bound to beads and typical Δ*ripA* infections elicit similar levels of IL-1β, even though the cells infected with beads should contain more bacteria initially (data not shown). Thus the decrease of IL-1β secretion during co-infections was not due to fewer Δ*ripA* infecting each cell. Together, these data demonstrate that active immune suppression mechanisms expressed by wild-type *F. tularensis* was sufficient to inhibit the inflammatory response induced by Δ*ripA*.

We also measured IL-1β secretion of Δ*pdpC* infected mBMDMs and found that cells infected with Δ*pdpC* bound to beads also had slightly increased IL-1β secretion when compared to wild-type infected cells (Figure 3). Co-infecting cells with wild-type and Δ*pdpC* bacteria resulted in decreased IL-1β secretion compared to Δ*pdpC* bacteria alone (Figure 3). Thus, Δ*pdpC* failed to entirely suppress the host immune response but suppression could be rescued by the presence of wild-type bacteria. Wild-type bacteria fully complemented suppression of IL-1β secretion during mixed infections of wild-type and Δ*pdpC* (p = 0.77) but there was a slight increase in the wild-type and Δ*ripA* mixed infection when compared to wild-type infections alone (p = 0.01). We hypothesize that the observed increase in IL-1β secretion during co-infections with Δ*ripA* is due to a small subset of cells that are infected by beads bound only to Δ*ripA* bacteria combined with the increased magnitude of IL-1β secretion observed during Δ*ripA* infections compared to Δ*pdpC* infections. However, we cannot rule out the possibility that Δ*ripA* may stimulate the immune response via a pathway that cannot be entirely suppressed by wild-type bacteria. In summary, co-infections of mutant and wild-type bacteria can rescue a phenotype caused by the inability of the mutant to properly modify the host cell.

### Phagosomal escape is functionally complemented during bead co-infections

**Figure 4:**
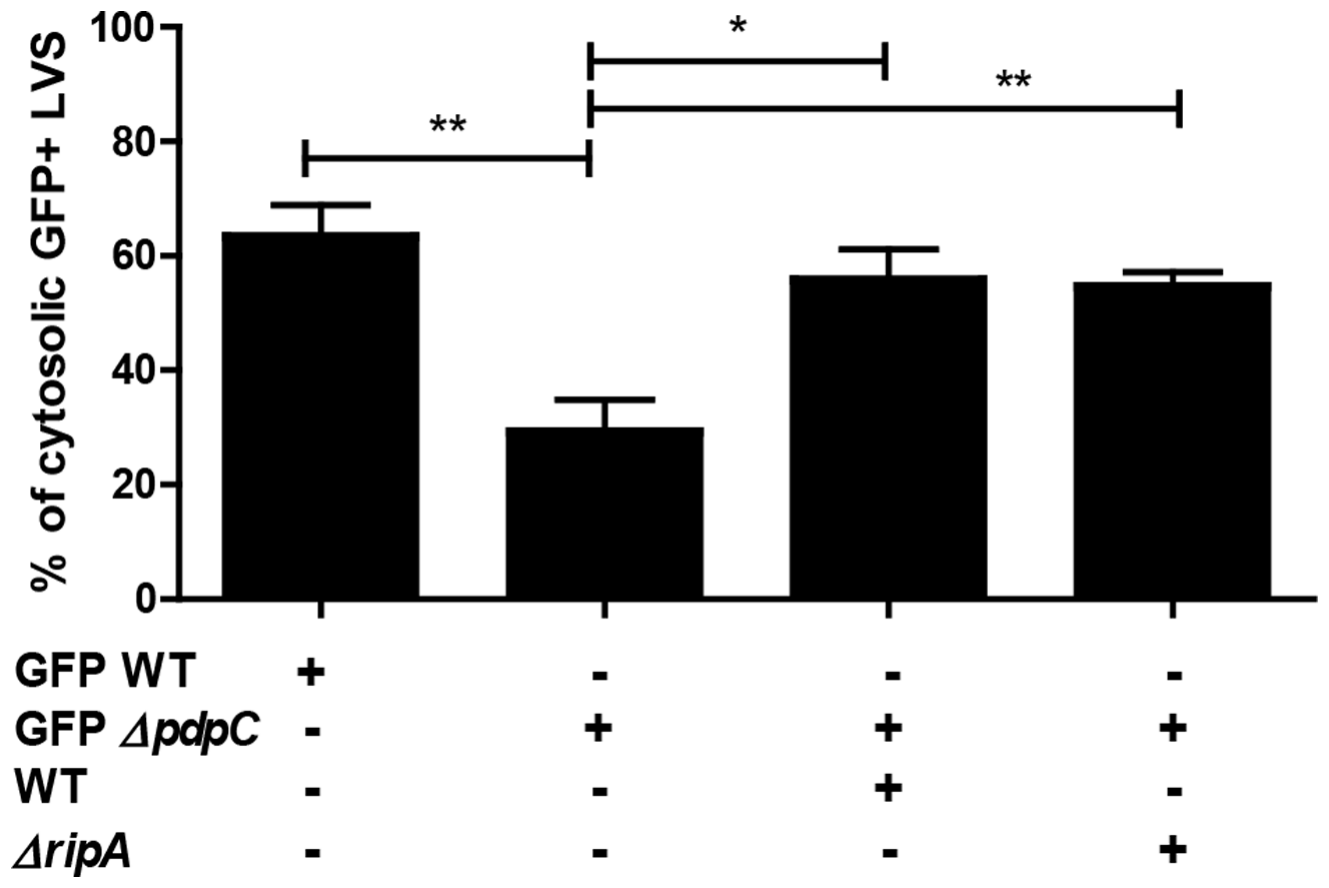
Functional trans-complementation via bead-bound bacteria complements phagosomal escape. J774 cells were inoculated with either GFP wild-type LVS or mixtures of GFP Δ*pdpC*, wild-type LVS, or Δ*ripA* (n = 4 independent experiments). The amount of cytosolic GFP positive bacteria was quantified by flow cytometry and normalized based on the amount of permeabilized cells, as determine by calnexin staining controls. All samples were bound to beads prior to infection. Data includes bacteria attached and detached from beads in the same sample. Bar graph represents the mean +/− SEM. * p<.05, **p<.01

Several FPI mutants, including Δ*pdpC*, as well as killed *F. tularensis* do not effectively escape the phagosome [5]–[13]. Therefore, *F. tularensis* phagosomal escape is a *F. tularensis* mediated process. As a result, wild-type bacteria should facilitate release of escape-defective mutants so long as the mutant and wild-type bacteria are within the same phagosome. To test this hypothesis, we bound Δ*pdpC* containing a GFP-expressing plasmid (GFPΔ*pdpC*) to beads and quantified the amount of GFPΔ*pdpC* present in the cytosol of J774 cells. We found that GFPΔ*pdpC* had reduced phagosomal escape at 2 hours post inoculation compared to GFP expressing wild-type bacteria and GFPΔ*pdpC* phagosomal escape was rescued by the presence of wild-type bacteria within the same phagosome (Figure 4). This result is consistent with other published analyses of Δ*pdpC* mutant phenotypes and indicates that PdpC contributes to, but is not absolutely required for, *F. tularensis* LVS phagosomal escape [5], [13]. More importantly, wild-type bacteria export the necessary effector protein(s) to allow both wild-type and Δ*pdpC* to escape the phagosome.

Bead co-infections achieve functional complementation of phagosomal escape across an entire population. This allows for the further characterization of phagosomal escape deficient mutants in the cytoplasmic environment. One caveat to using wild-type bacteria for complementation is that wild-type bacteria may out-compete certain mutants in the cytosol or obscure additional host-pathogen interactions. We hypothesized that any mutant strain that escapes the phagosome can be used to complement phagosomal escape of escape deficient mutants. To test this hypothesis, we co-infected cells with GFPΔ*pdpC* and Δ*ripA*. The Δ*ripA* strain escapes the phagosome with similar kinetics as wild-type bacteria, but does not replicate inside the host cell [14]. Indeed, Δ*ripA* functionally complemented GFPΔ*pdpC* phagosomal escape when GFPΔ*pdpC* entered through the same phagosome as Δ*ripA* (Figure 4). Our data demonstrate that infecting cells with Δ*pdpC* and a phagosome escape competent *F. tularensis* bacterium results in phagosomal escape of both bacteria. Thus, pairing a phagosomal escape deficient mutant with an escape competent mutant will allow for further characterization of cytoplasmic phenotypes associated with the phagosomal escape deficient mutant.

### Bead co-infections complement intracellular survival

*F. tularensis* mutants can be deficient for intracellular survival or replication due to the inability of the mutant to perform a required interaction with the host or due to an intrinsic intracellular survival or replication defect. Co-infections with both mutant and wild-type bacteria can be used to determine whether the mutant fails to properly control a host-pathogen interaction

**Figure 5:**
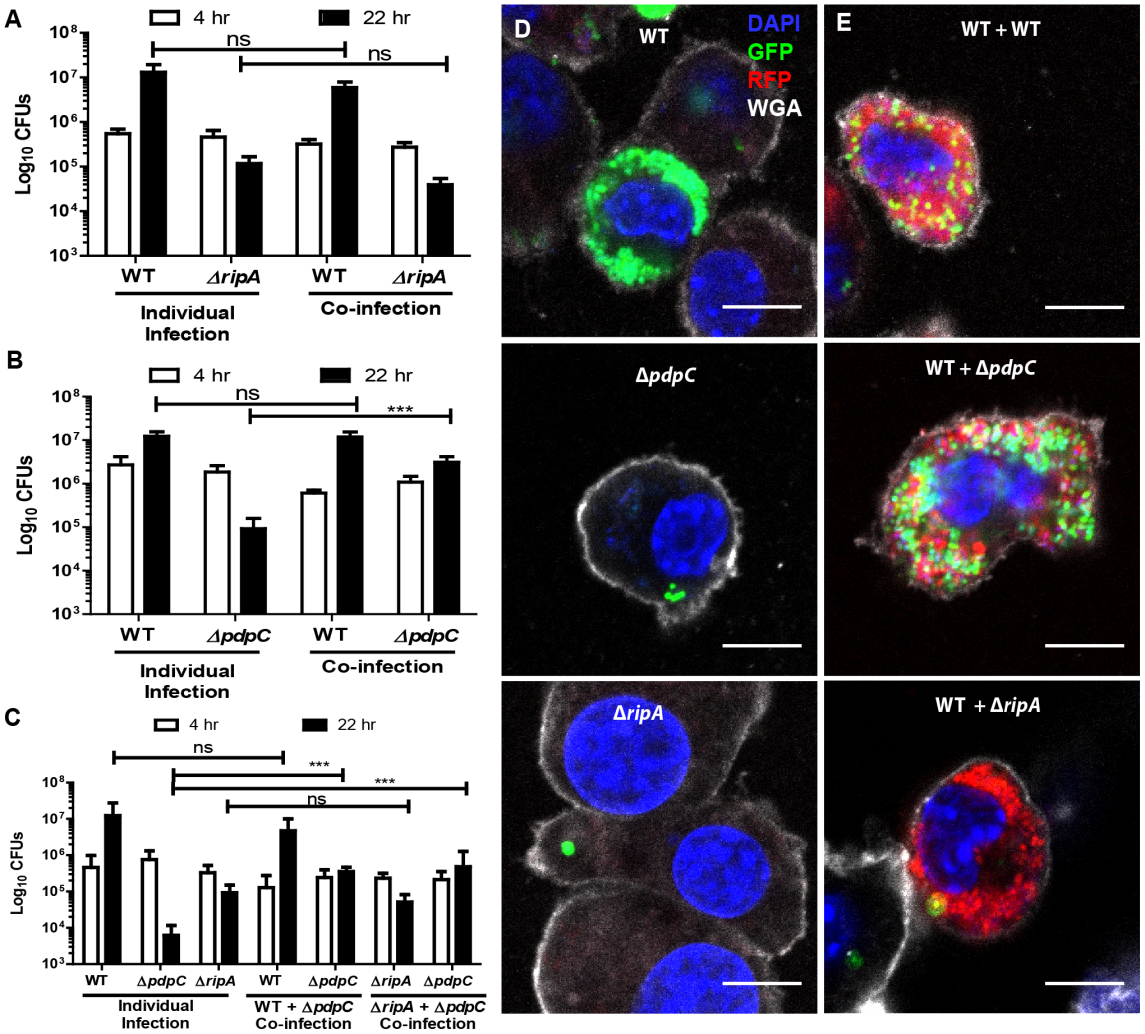
Functional trans-complementation via bead-bound bacteria complements intracellular proliferation of Δ*pdpC* but not Δ*ripA*. (**A**) Kanamycin resistant wild-type LVS or hygromycin resistant Δ*ripA* were individually or co-inoculated into J774 cells and assayed for intracellular proliferation at 4 and 22 hours post inoculation. (**B**) Kanamycin resistant wild-type LVS or hygromycin resistant Δ*pdpC* were individually or co-inoculated into J774 cells and assayed for intracellular proliferation at 4 and 22 hours post inoculation. (**C**) Kanamycin resistant wild-type LVS, kanamycin resistant Δ*ripA* or hygromycin resistant Δ*pdpC* were individually or co-inoculated into J774 cells and assayed for intracellular proliferation at 4 and 22 hours post inoculation. Results are from 3 independent experiments performed in duplicate or triplicate. Bar graphs represent the mean +/− the standard deviation. (**D**) Representative fluorescence micrographs of J774 cells inoculated for 22 hours with GFP wild-type, GFPΔ*pdpC* or GFPΔ*ripA* bacteria attached to beads. (**E**) Representative fluorescence micrographs of J774 cells inoculated with beads bound to DsRed LVS and either GFP WT, GFPΔ*pdpC* or GFPΔ*ripA*. Blue represents the nucleus (DAPI), green represents the indicated GFP LVS mutants, red represents DsRed wild-type LVS, and white represents the plasma membrane stain wheat germ agglutinin (WGA). All scale bars represent 10 µm. All samples were bound to beads prior to infection. Not significant (ns), p>.05, * p<0.05, ***p<0.005.

The *ripA* gene encodes a hypothetical protein of unknown function that is required for *F. tularensis* intracellular proliferation, likely through regulation of lipid A synthesis [14] (our unpublished data). We found that co-infecting J774 cells infected with wild-type *F. tularensis* did not complement Δ*ripA* intracellular growth (Figure 5A). Likewise, individual cells had similar numbers of Δ*ripA* expressing GFP regardless of whether or not wild-type *F. tularensis* was present in the same cell (Figure 5D, 5E). These data further indicate that *ripA* is involved in an intrinsic bacterial process essential for intracellular bacterial proliferation.

The PdpC protein is encoded on the FPI and Δ*pdpC* bacteria are deficient for intracellular proliferation [5], [13]. Intracellular proliferation of Δ*pdpC* is not consistent across the entire population, as this strain replicates to high numbers in a small subset of cells [13]. Since the FPI is proposed to encode a secretion system, the failure of FPI gene deletion strains to replicate in the host cell is likely due to the inability to initiate host-pathogen interactions [19]. Thus Δ*pdpC* replication may be rescued by the presence of wild-type bacteria. Consistent with previous reports, the amount of viable intracellular LVS Δ*pdpC* bacteria decreased over time (Figure 5B) [5], [13]. On average, the number of Δ*pdpC* organisms decreased over 2 orders of magnitude between 4 and 22 hours post inoculation (9 independent experiments). However, when J774 cells were co-infected with a mixture of wild-type and Δ*pdpC* bound to beads, Δ*pdpC* survival was functionally complemented by the wild-type bacteria (Figure 5B). Specifically, we observed a slight increase in Δ*pdpC* replication during co-infections with wild type between 4 and 22 hours (2.0+/−0.3 fold, n = 7 independent experiments, p = 0.0014) (Figure 5B, 5D, 5E). We conclude that wild-type bacteria complemented the defect of Δ*pdpC* by secreting the effector(s) necessary to manipulate the host cell into being permissive for *F. tularensis* survival. Thus, *F. tularensis* LVS Δ*pdpC* does not survive inside of J774 cells due to its inability to properly manipulate an interaction(s) with the host that *F. tularensis* requires for intracellular survival.

Although Δ*pdpC* replicated slightly when wild-type bacteria were present in the same cell, Δ*pdpC* replication did not achieve wild-type levels. This is interesting because Δ*pdpC* escaped the phagosome, albeit at lower levels than wild-type, but was still inhibited by the host cell during individual infections (Figure 4, 5B) [5], [13]. Thus, wild-type bacteria may promote Δ*pdpC* survival in the host cytosol (Figure 5B) but wild-type *F. tularensis* either out-competes Δ*pdpC* or *pdpC* is also required for a cell intrinsic process to fully restore intracellular replication. To distinguish between these possibilities, we co-infected cells with Δ*pdpC* and Δ*ripA*. The Δ*ripA* strain has an intact FPI and rescues Δ*pdpC* phagosmal escape, but since Δ*ripA* does not replicate, it should not out-compete Δ*pdpC*. We found that co-infections with Δ*ripA* and Δ*pdpC* resulted in Δ*pdpC* survival but proliferation did not increase to wild-type levels (Figure 5C). Thus, we conclude that PdpC is required for intracellular survival through a host-mediated process but has a secondary function that enhances bacterial proliferation.

Together, the Δ*pdpC* and Δ*ripA* intracellular replication data demonstrate that co-infecting cells with wild-type and mutant bacteria can differentiate between mutants that fail to replicate due to defective host-pathogen interactions and mutants that are defective for growth due to intrinsic replication defects.

## Discussion

We developed and optimized a method to reliably deliver two distinct *F. tularensis* bacteria into the same host cell. Co-infection of cells with bacteria bound to beads allows for functional complementation of host-pathogen interactions, which can be used to identify mutants that fail to induce a host-pathogen interaction or to complement a specific host-pathogen interaction.

The Δ*ripA* strain fails to replicate inside of cells and induces IL-1β secretion [2], [14], [15]. With the described co-infection method, neighboring wild-type bacteria functionally complement IL-1β suppression in Δ*ripA* infected cells but not Δ*ripA* intracellular proliferation. The *Francisella novicida* Δ*ripA* strain has also been shown to have increased intracellular lysis compared to wild-type *F. novicida* bacteria, which leads to increased cytokine secretion via the AIM2 inflammasome [20]. Herein we show that wild-type *F. tularensis* LVS bacteria can suppress cytokine secretion in cells infected with Δ*ripA* bacteria. Assuming that RipA has an identical function between the different *Francisella* species, these data indicate that wild-type LVS bacteria, but not Δ*ripA*, suppress the AIM2 inflammasome.

Interestingly, wild-type *F. tularensis* bacteria replicated in cells that contained Δ*ripA* bacteria, which indicates that the cell cytosol remains permissive for *F. tularensis* replication and that the proliferation defect is specific to Δ*ripA* bacteria. These data also indicate that increased cytokine secretion and intracellular proliferation phenotypes seen in Δ*ripA* infections occur through different mechanisms because one phenotype is functionally complemented while the other is not. Additionally, we were able to genetically distinguish between Δ*ripA* cytokine suppression and intracellular replication. A chromosomal *ripA* N21A point mutant of LVS proliferated in the cytosol at a rate comparable to wild-type bacteria, but failed to suppress IL-1β secretion (data not shown). Taken together, the described co-infection method indicates that RipA is involved in cytokine suppression (a host-pathogen interaction) and a separate, intrinsic bacterial process for intracellular replication.

The Δ*pdpC* strain was functionally complemented for phagosomal escape, intracellular survival, and IL-1β suppression. Thus, PdpC contributes to those host-pathogen interactions. Interestingly, Δ*pdpC* replication never reached the same level as wild-type bacteria during co-infections even though Δ*pdpC* escaped the initial phagosome with the wild-type bacteria. These data hint that PdpC is involved in an intrinsic bacterial replication process that is required for optimal growth. It is also possible that a local host-pathogen interaction is required that is not reliably complemented by neighboring bacteria. Further investigation is needed to determine if the intracellular growth defect is specific to Δ*pdpC* or if other FPI genes are required for replication in the host cell cytosol.

The antibody used throughout these experiments has been used to identify *F. tularensis* Schu S4 by microscopy and for purifying Schu S4 from infected cell lysates [21], [22]. As described, this method should be compatible with a range of *F. tularensis* species with O-antigen structures similar to *F. tularensis* LVS. More importantly, the described co-infection method is compatible with any biotinylated antibody, so antibodies specific to surface molecules of *F. tularensis* novicida or other bacterial species can be used to link bacterial cells to beads.

Binding bacteria to beads does alter some aspects of infection that must be taken into consideration when designing experiments. The beads are dense and sink to the bottom of the well, allowing for a substantially lower multiplicity of infection (MOI) while maintaining a high infection frequency. More bacteria are phagocytosed per cell when bacteria are bound to beads compared to a typical infection. This may require time points to be taken earlier than a typical infection and the magnitude of certain phenotypes, particularly intracellular proliferation, are slightly different. Lastly, we expect that phagocytosis of beads coated in bacteria will primarily occur through typical phagocytic routes of a given bacteria because bacteria on the bead surface still interact with the cell. But phagocytosis may occasionally occur through a different mechanism than in a typical infection. For example, some Fc receptor mediated phagocytosis likely occurs in some cells due to antibodies bound to the beads. Despite these potential pitfalls, our individual infection controls for IL-1β secretion, phagosomal escape, and intracellular proliferation resulting from infection with bead-bound bacteria are consistent with previously published data based on infection by free bacterial cells [2], [5], [13], [14].

Bead co-infections provide a consistent method of functionally complementing most intracellular bacterial manipulations of the host cell. However, there are a few specialized cases in which further characterization beyond bead co-infections is necessary. We expect that some gain of function mutants and host-pathogen interactions that require local manipulation will not be reliably complemented. For example, immune signaling phenotypes that are not complemented by wild-type bacteria are likely gain of function mutations because the mutants stimulate the immune system in a manner that wild-type bacteria cannot suppress. Likewise, xenophagy evasion and re-entry into *Francisella* containing vacuoles likely requires local host-pathogen interactions. As a result, a lack of complementation for mutants that are strongly suspected of manipulating the host cell can also narrow down potential functions for a given protein. Altogether, bead co-infections are a reliable method to gain insight into the function of a gene of interest, identify mutants that fail to initiate a host-pathogen interaction, and to aid in downstream experimental design.

## Materials and Methods

### Ethics Statement

All animal studies were approved by and conducted according to the Institutional Animal Care and Use Committee (NIH/PHS Animal Welfare Assurance Number: A3410-01, USDA Animal Research Facility Registration Number: 55-R-004, AAALAC Institutional Number: #329) guidelines of the University of North Carolina- Chapel Hill (IACUC ID 13-213).

### Bacteria and plasmids

*Francisella tularensis* subsp *holartica* LVS was obtained from the CDC Atlanta, GA. A *pdpC* deletion construct was made by splice overlap extension (SOE) PCR as described previously [14]. Primers were used to delete all but the first five amino acid (MNDKY) and the last five codons (KISS stop) while keeping the deletion in frame. After blunt end cloning into pCR BLUNT II (Invitrogen), the SOED *pdpC* fragment was removed by BamHI-Not I digest and cloned into suicide vector pMP812 [23]. Integrants were selected on kanamycin (10 ug/ml). Resolved integrants were selected by growth overnight in brain heart infusion (BHI) broth (BD Biosciences) supplemented with isovitalex followed by plating on chocolate containing 10% sucrose. Resolved integrants were sequenced to confirm in-frame deletion of *pdpC* and integrity of the flanking sequence. Deletion of both *pdpC* genes was verified by PCR. Genetic complementation of *pdpC* by constitutively expressing the *pdpC* gene on pMP822 restored intracellular proliferation (Figure S1) [24]. The Δ*ripA* in-frame deletion and GFPΔ*ripA* strains were previously generated [25].

GFP wild-type LVS (GFP-wt) was generated by Hall et al [26] and GFP-Δ*pdpC* was generated using the same GFP plasmid. DsRed LVS and luciferase expressing LVS were generated with the plasmids from DsRed Schu S4 and luciferase expressing Schu S4 respectively [22]. Hygromycin B resistant Δ*pdpC* and Δ*ripA* were generated by transforming the deletion mutant bacteria with the hygromycin B resistance plasmid pMP831 [24]. The kanamycin resistant wild-type strain was generated by transforming wild-type bacteria with the kanamycin resistance plasmid pMP828 and kanamycin resistant Δ*ripA* with the pkkMCS plasmid [14], [24].

### Cell Culture

J774A.1 Macrophage like cells were obtained from ATCC and grown in DMEM with 4.5 g/L glucose and supplemented with 10% FBS, L-glutamine and sodium pyruvate (all from Gibco). Mouse bone marrow derived macrophages were generated from a C57Bl/6 as previously described [27].

### Binding *F. tularensis* to the magnetic beads

800 nm streptavidin coated magnetic beads (Solulink) were blocked with sterile filtered Tris buffered saline (TBS) containing 0.1% casein for 20 minutes. The beads were washed 4 times with antibody wash buffer (100 mM Tris, 150 mM NaCl and 0.05% tween 20 in distilled water, pH 8.0). For every 10 ug of beads, we added 2.5 µg of anti-*Francisella tularensis* LPS antibody (US Biological) that was previously biotinylated using a FluoReporter Mini-Biotin-XX Protein Labeling Kit (Invitrogen) following the manufacturer's instructions. The antibody was suspended in antibody wash. After 30 minutes of rocking at 4°C, the beads were washed twice in antibody wash buffer and twice in PBS. 10 µg of magnetic beads coated with anti- LPS antibody were mixed with 8×10^8^ bacteria for 20 minutes. Each sample was then washed twice in PBS to remove unbound *F. tularensis*. After the final wash, the beads were suspended in cell culture media and ready to use for the given experiment. All washes were performed by placing a 12×75 mm round bottom tube containing the sample on a BD IMagnet (BD biosciences) and waiting approximately 2 minutes for the beads to move toward the magnet. After all wash steps, approximately 1×10^7^ beads bound to bacteria were present for every 10 ug of beads initially added (based on plating bacteria bound to beads on chocolate agar). The amount varied up to 4×10^6^ beads between experiments. All experiments were inoculated assuming 1×10^7^ bacteria bound to beads per 10 ug of starting beads.

For samples where multiple strains were added together, 4×10^8^ of each strain was added. For all mixed infections, the inoculum of each type of bacteria was plated and compared to ensure both types of bacteria were equally represented. Typically the ratio of one type of bacteria to the other was between 1: 0.8 and 1: 1.2 (data not shown). Infecting cells at a MOI higher than 1 can result in each cell taking up a substantial number of beads, which may impact results (data not shown). Bead aggregates can form over time which may impact the number of bacteria that enter each cell.

### Microscopy

For micrographs of bacteria on beads, GFP-LVS and/or DsRed labeled LVS were coated onto the beads as described above. The bacteria bound beads were fixed in 4% paraformaldehyde and were placed into an 8 well chamber slide (Nunc). The beads were allowed to settle and the fixative was carefully removed.

For images of infected cells, beads were prepared as above with the indicated bacteria. J774 cells were inoculated for 2 hours and then the media was removed and replaced with media containing 25 µg/ml of gentamicin. The cells were washed and fixed with 4% paraformaldehyde at 4 or 22 hours post inoculation. Cells were treated with 50 mM ammonium chloride for 10 minutes and then stained with 10 µg/ml of AF647 wheat germ agglutinin (Invitrogen) where indicated.

All samples were mounted using a DAPI containing mounting media (Vector Shield). Images were acquired using a Zeiss 700 confocal laser scanning microscope (Zeiss) using Zen image acquisition software (Zeiss) or an Olympus FV500 confocal scanning laser microscope (Olympus). Images were cropped and scale bars were added using ImageJ [28].

### Flow Cytometry

To assess the frequency of both types of bacteria binding to a single bead, beads were prepared as above with GFP-LVS and/or CellTrace Far Red DDAO-SE (Invitrogen) labeled wild-type LVS. Immediately following the final wash, the beads were stained with pacific blue conjugated anti- *F. tularensis* LPS antibody (US biological) (Pacific blue antibody labeling kit [Invitrogen] following the manufacturers protocol). The beads were washed once more and then immediately fixed using 4% paraformaldehyde. The event trigger on the flow cytometer was set to only record Pacific Blue positive events.

Phagosomal escape assays were performed as previously described [29]. Briefly, J774 cells were inoculated with beads coated with the indicated wild-type or deletion mutants bacteria (prepared as above). 2 hours post inoculation, the cells were suspended and stained with pacific blue conjugated anti- *Francisella tularensis* LPS antibody to label extracellular bacteria. After the antibody was washed away with KHM buffer, the cells were permeabilized with 100 ul of 10 µg/ml digitonin and the cells were stained with AF647 conjugated anti- *Francisella tularensis* LPS antibody to stain cytosolic bacteria. The cells were lysed and the samples were then analyzed by flow cytometry. We compared the ratio of intracellular cytosolic bacteria (GFP+ AF647+ Pac Blue-) to bacteria in the phagosome (GFP+ AF647- Pac Blue-). The data from these experiments includes both bacteria bound to beads and unattached bacteria. No significant difference in localization was seen between *F. tularensis* attached to beads and unbound bacteria, although bacteria bound to beads tended to escape the phagosome at higher rates. Control cells were stained with calnexin to assay the percentage of cells that were permeabilized. The percentage of permeabilized cells was used to determine the total percentage of bacteria that escaped the phagosome.

To assay for bacterial dissociation from the beads prior to inoculation, GFP-LVS was bound to beads as previously described and stained with pacific blue conjugated anti- *F. tularensis* LPS antibody. The fixed sample was then analyzed for the percentage of bacteria bound to beads. To assess the amount of dissociation from beads inside of cells, GFP-LVS was bound to beads as previously described. The cells were removed from the plate, washed in KHM buffer [29], and permeabilized with digitonin 2 hours post inoculation. The permeabilized cells were stained with an AF647 conjugated anti- *F. tularensis* LPS antibody (US biological). The antibody was washed away and then the infected cells were lysed by vortexing the cells in distilled water. AF647+, GFP+ events were analyzed for attachment to a bead based on size.

All samples were analyzed using a Cyan Flow Cytometer (Dako) with the event trigger set to record only pacific blue positive events or GFP positive events depending on the experiment. All histograms were pre-gated so that only single events were analyzed.

### Intracellular bacterial proliferation assay

The indicated LVS mutants were coated on beads as previously described. J774 cells were seeded at approximately 2.5×10^5^ cells per well the night before infection and were infected with an approximate multiplicity of infection (MOI) of 1 bead per cell (assuming 5×10^5^cells prior to infection). The media was replaced with media containing 25 µg/ml of gentamicin 2 hours post inoculation. At 4 and 22 hours post inoculation, the cells were scraped off the plate, lysed, serially diluted and plated on chocolate agar containing isovitalex and either kanamycin or hygromycin to assess bacterial proliferation. As expected, hygromycin resistant colonies did not form under kanamycin selection and vice versa (data not shown).

### Quantification of bacteria per bead

*F. tularensis* LVS containing a luciferase –expressing plasmid was bound to beads following the previously described method. Half of the sample was plated on chocolate agar to assay for the number of beads bound to bacteria and the other half was placed into an Infinite M200 Pro plate reader (Tecan) and the amount of light produced was quantified and compared to a standard curve consisting of know quantities of *F. tularensis* LVS containing the luciferase plasmid.

The number of bacteria per cell was determined based on the results at 4 hours post inoculation of the wild-type bacteria from the intracellular proliferation assay described previously. We assumed that 60% of the cells were infected based on flow cytometry of similar samples examined 2 hours post inoculation. Since gentamicin is added 2 hours post inoculation, additional cells should not be infected. We also assumed that the J774 cells doubled overnight, resulting in 5×10^5^ cells, that each cell was infected with 1 bead, that 100% of bacteria were bound to beads and no bacterial death or replication occurred between 0 and 4 hours post inoculation.

### IL-1β ELISA

Murine bone marrow derived macrophages were seeded at 5×10^5^ cells per well in 24-well plates. Beads coated with the appropriate bacterial strains and were used to inoculate the cells at an MOI of approximately 1 bead per cell. The cell supernatant was collected 22 hours post inoculation and the amount of IL-1β was measured using a BD OptEIA mouse IL-1β ELISA set (BD biosciences) following the manufacturers protocol.

### Statistics

All statistics were performed using an unpaired Student t-test using the compiled data from all experiments performed. Intracellular proliferation assays were log_10_ transformed prior to statistical analysis.

## Supporting Information

Figure S1 **Genetic complementation of Δ** ***pdpC*** **intracellular proliferation.** Intracellular proliferation assay of J774 cells infected with wild-type, Δ*pdpC* or Δ*pdpC* with a complementation plasmid containing the *pdpC* gene. The bar graph represents the mean +/− the standard deviation. Data is a compilation of 3 independent experiments performed in triplicate. ***p<0.005
(TIF)
