# Supplementary figures and images for: A Method for Functional Trans-Complementation of Intracellular Francisella tularensis
Source: PLoS One. 2014 Feb 4;9(2):e88194. doi: 10.1371/journal.pone.0088194 (PMC3913766; doi:10.1371/journal.pone.0088194)

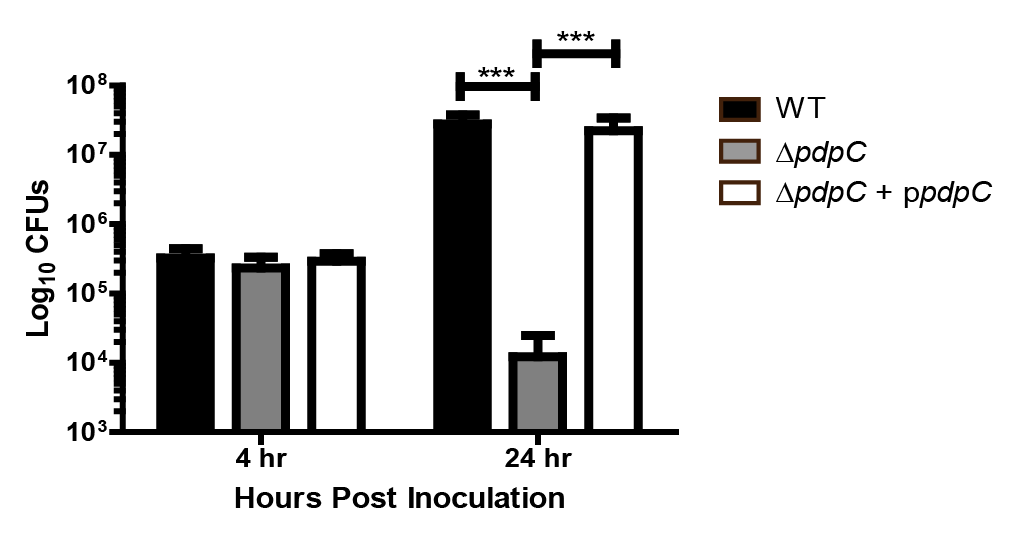

Supplement: Figure S1 — Genetic complementation of Δ pdpC intracellular proliferation. Intracellular proliferation assay of J774 cells infected with wild-type, ΔpdpC or ΔpdpC with a complementation plasmid containing the pdpC gene. The bar graph represents the mean +/− the standard deviation. Data is a compilation of 3 independent experiments performed in triplicate. ***p<0.005 (TIF) [file pone.0088194.s001.tif]
